# Case report: Limb-sparing surgery of tibial chondrosarcoma with frozen autologous bone graft using liquid nitrogen in a dog

**DOI:** 10.3389/fvets.2023.966513

**Published:** 2023-04-03

**Authors:** Masakazu Shimada, Tomokazu Nagashima, Masaki Michishita, Daisuke Yazawa, Yasushi Hara

**Affiliations:** ^1^Division of Veterinary Surgery, Department of Veterinary Science, Faculty of Veterinary Medicine, Nippon Veterinary and Life Science University, Musashino, Tokyo, Japan; ^2^Division of Veterinary Pathology, Department of Veterinary Science, Faculty of Veterinary Medicine, Nippon Veterinary and Life Science University, Musashino, Tokyo, Japan

**Keywords:** chondrosarcoma, limb-sparing surgery, liquid nitrogen autologous graft, stifle arthrodesis, dog

## Abstract

Chondrosarcoma is the second most common primary bone tumor after osteosarcoma in dogs. Chondrosarcoma has a good prognosis owing to its low metastatic rate and long survival time, even with amputation alone. However, amputation risks reducing the quality of life in patients with other orthopedic diseases of the non-affected limb, neurological diseases, or large body size. Limb-sparing surgery with frozen autologous bone grafting using liquid nitrogen allows bone quality to be maintained in the normal bone area while killing tumor cells, thereby preserving the affected limb. Thus, it is expected to maintain the quality of life. We describe herein limb-sparing surgery for tibial chondrosarcoma with frozen autologous bone graft using liquid nitrogen in an 8-year and 8-month-old castrated male bulldog weighing 29.2 kg. The patient had chondrosarcoma of the left tibia, suspected cranial cruciate ligament rupture of the right stifle, and degenerative lumbosacral stenosis. In such a case, amputation would increase the burden on the non-affected limb or spine, which could cause difficulty in walking; therefore, we performed limb-sparing surgery. Postoperatively, although a circumduction gait associated with stifle arthrodesis remained, the patient maintained the quality of life for 20 months, and the owner was satisfied with the results.

## 1. Introduction

Chondrosarcoma (CSA) is the second most common primary bone tumor after osteosarcoma, but its incidence is low at ~10% (1, 2). CSA most commonly occurs in the nasal cavity (28.8%) and limbs (17.5%) (3). Previous reports have shown that CSA has a longer survival time and lower metastasis rate than osteosarcoma (1).

Limb-sparing surgery can maintain quality of life (QOL) in patients expected to have significant difficulty walking after limb amputation, such as patients with other orthopedic diseases in the unaffected limb, patients with neurological diseases, or patients with large body size (4, 5). There are several techniques for limb-sparing surgery in veterinary medicine; these include allogeneic cortical bone grafting, endoprosthesis, stereotactic radiosurgery and fixation, and lateral manus translation (6–9). However, there may be limitations in the availability of allogeneic cortical bone and implants to be used at different hospitals and limitations in biomechanical strength.

The reuse of parts of bone tumors has been reported in human medicine. In recent years, limb-sparing surgery using liquid nitrogen freezing, which is readily available and minimizes the loss of bone strength, has been reported for bone tumors (10, 11). This surgery is expected to have good osteoconductive and osteoinductive capabilities with less adverse immune responses because autologous bone can be used. However, reports on the use of liquid nitrogen for freezing bone tumors in animals are rare but have been reported for the scapula in a cat and the mandible in a dog (12, 13). However, there have been no long-term postoperative observations of CSA reported to date. The burden on the unaffected limbs after amputation is a particular concern because CSA has a promising long-term outcome, which makes a focus on the QOL of the patients important.

## 2. Case description

**Figure 1:**
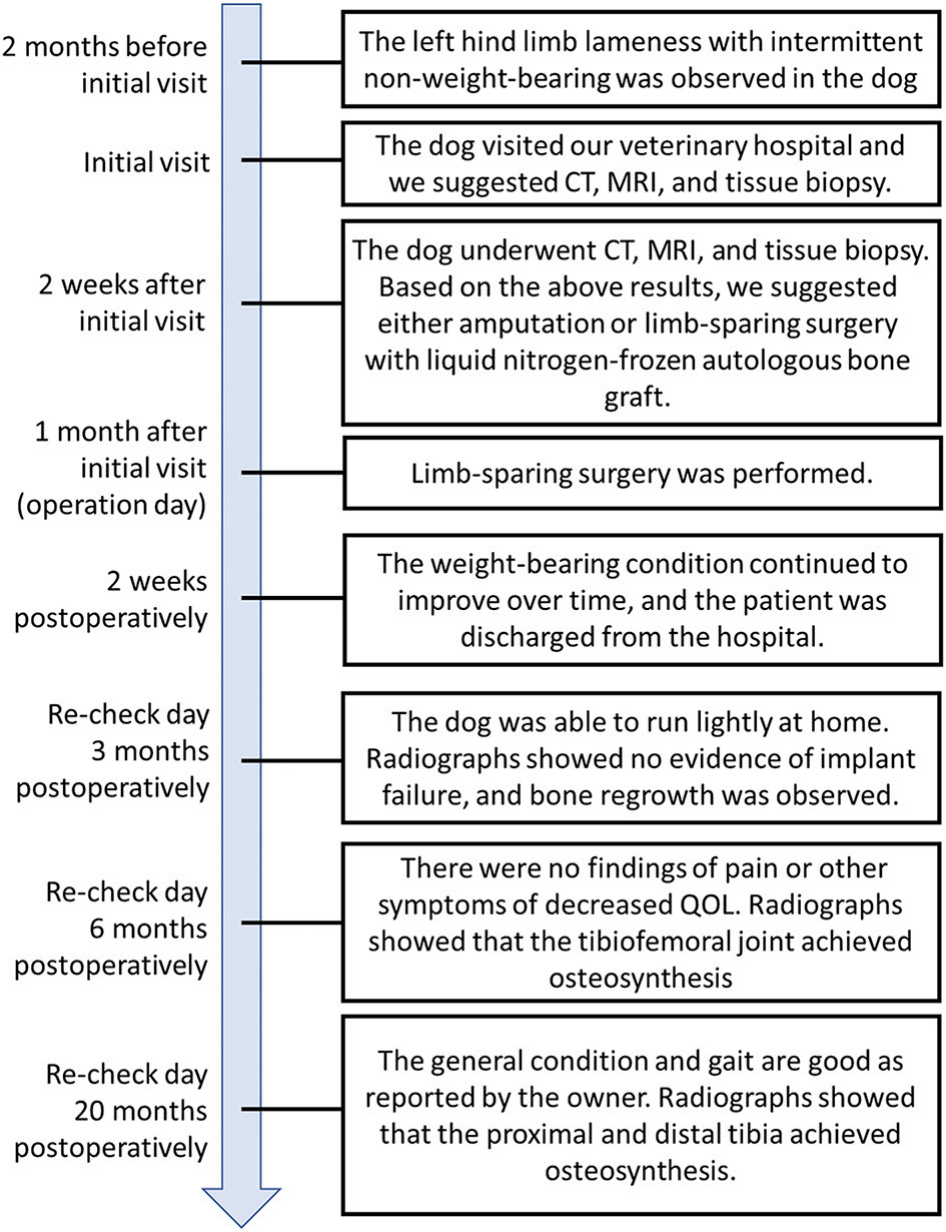
Timeline with relevant data from the episode of care. CT, computed tomography; MRI, magnetic resonance image.

**Figure 2:**
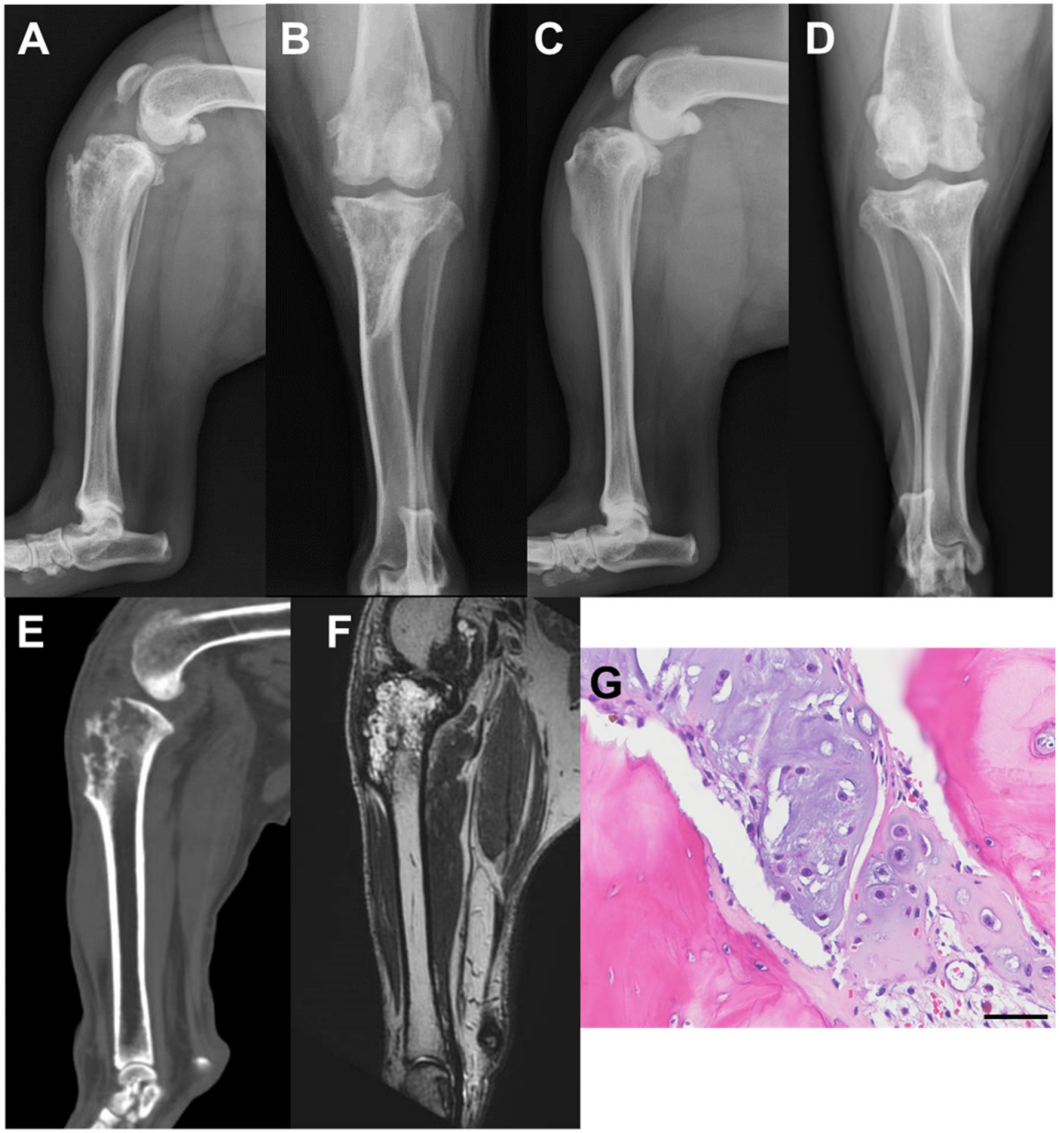
Radiographic images of the stifle joints at the time of initial examination: computed tomography (CT), magnetic resonance (MR), and histological images. **(A)** Mediolateral image of the left stifle; **(B)** Craniocaudal image of the left stifle; **(C)** Mediolateral image of the right stifle; **(D)** Craniocaudal image of the right stifle; **(E)** The left stifle joint midsagittal section on CT; **(F)** The left stifle joint midsagittal section in MR T2-weighted image; **(G)** Microscopic findings of hematoxylin-eosin staining (scale bar: 50 μm).

An 8-year, 8-month-old castrated male bulldog weighing 29.2 kg was referred to our hospital with a primary complaint of lameness with intermittent inability to bear weight in the left hind limb for 2 months (Figure 1). The patient had also presented with lameness in the right hind limb 5 months prior that subsequently improved without treatment. The patient was able to walk in the hospital but did not want to walk for a long time. On palpation, cranial drawer and tibial compression tests of the left stifle joint were positive, and the stifle joint showed pain on extension. The cranial drawer and tibial compression tests of the right stifle joint were negative. In addition, lordosis test showed a painful reaction, and both hind limbs showed decreased conscious proprioception. Radiography revealed increased radiolucency of the proximal left tibia (Figures 2A, B). The right stifle showed mild cranial displacement of the tibia and findings suggestive of osteoarthritis (Figures 2C, D). The findings of the thoracolumbar radiograph were indicative of spondylosis deformans in several vertebrae, including the lumbosacral vertebrae. Chest radiography revealed no findings suggestive of metastasis. Based on these results, the left stifle was suspected to have a bone tumor based on bone resorption of the proximal tibia, and the right stifle was suspected to have a ruptured cranial cruciate ligament associated with osteoarthritis. Degenerative lumbosacral stenosis was also suspected in the lumbosacral joint. As such, concurrent computed tomography (CT), magnetic resonance imaging (MRI), and tissue biopsy of the left tibia, and MRI of the lumbosacral joint were recommended.

In this case, CSA was suspected as a result of biopsy. Subsequently, we report CSA of the proximal tibia treated with limb-sparing surgery using an autologous bone graft after freezing by liquid nitrogen and stifle arthrodesis that resulted in a favorable prognosis.

CT showed bone resorption in the left proximal tibia (Figure 2E). In addition, the same region showed a high signal intensity in the MR T2-weighted images (T2WI; Figure 2F). Evaluation of the lungs revealed no findings suggestive of tumor metastasis, even on CT. The intervertebral foramen of the lumbosacral joint was bilaterally narrowed during the extension on T2WI. Although tissue biopsy revealed a suspected chondroma or CSA, a definitive diagnosis could not be made due to the small amount of material collected (Figure 2G). Based on the above results, we suggested either amputation or limb-sparing surgery with liquid nitrogen-frozen autologous bone graft, and the owner requested limb-sparing surgery.

Surgery was performed one month after the initial visit. Anesthesia was induced with propofol [4 mg/kg intravenous (IV)] and midazolam hydrochloride (0.3 mg/kg IV), and endotracheal intubation was performed. Anesthesia was maintained with isoflurane and oxygen, and a ventilator ensured respiratory management. After induction of anesthesia, 0.5% bupivacaine (0.2 ml/kg) and morphine (0.1 μg/kg) were administered epidurally for analgesia, and continuous infusion of remifentanil (5–15 μg/kg/h) was administered intraoperatively. Cefmetazole sodium (25 mg/kg, IV) antibiotic was first administered ~1 h before the incision and at 90-min intervals during the perioperative period (14).

**Figure 3:**
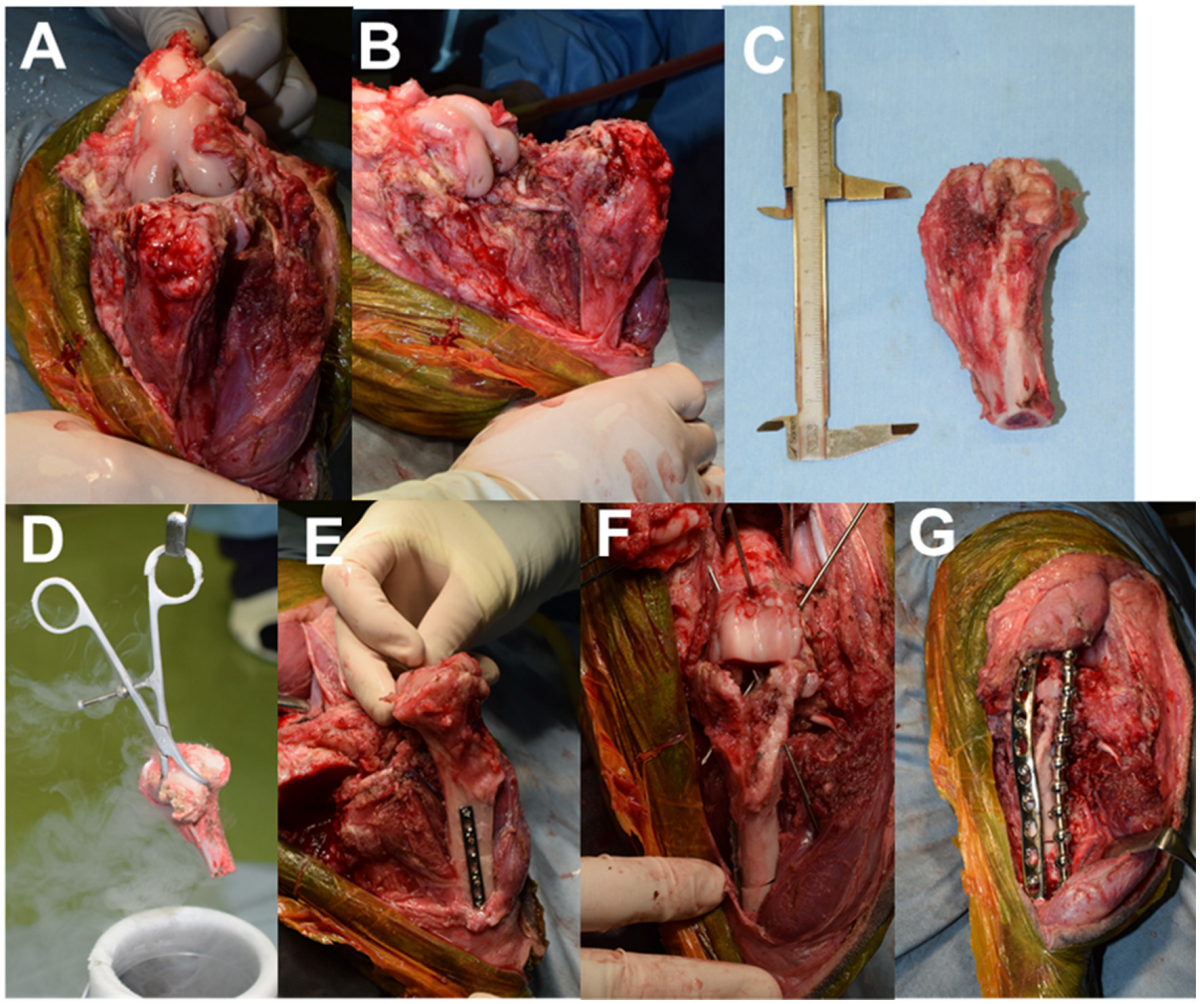
Intraoperative images. **(A)** Cranial view after removing soft tissue around the tibia; **(B)** Medial view after removing soft tissue around the tibia; **(C)** Resected proximal tibia; **(D)** proximal tibia frozen using liquid nitrogen; **(E)** Tibial tuberosity collapsed after freezing. **(F)** Fixation of the tibiofemoral joint with K-wire; **(G)** Arthrodesis with two plates.

The stifle joint was approached laterally. A skin incision was made from the greater trochanter to the tarsal joint along the femur and tibia. After incising the joint capsule, the ligaments (cruciate ligament, collateral ligament, and meniscofemoral ligament) and tendons (tendons of the long digital extensor and popliteus) of the tibiofemoral joint were removed from the femoral attachment. The patellar tendon was resected at the patellar apex (Figures 3A, B). The muscles around the proximal tibia were dissected off, and the tibia was osteotomized ~3 cm distal to the area of high signal intensity on T2WI (Figure 3C). Tissue was collected from the medullary cavity of the distal end of the proximal tibial fragment and a portion of the tumor lesion for histological diagnosis. Thereafter, the removed proximal tibia was immersed in liquid nitrogen for 20 min, at room temperature for 15 min, and in a 30°C-physiological saline solution for 15 min, according to previous reports (Figure 3D) (10). The subsequent process resulted in the collapse of the tumor lesion (tibial tuberosity) (Figure 3E). After osteotomy of the proximal articular surface of the tibia and distal articular surface of the femur, stifle arthrodesis was performed as 135 degrees of the femorotibial joint angle. A 2.0-mm diameter dynamic compression plate (Mizuho Medical Co., Ltd., Tokyo, Japan) was used to temporarily fix the proximal and distal fragments of the tibia. Subsequently, the cross-pin method was used to fix the tibiofemoral joint using a 1.6-mm diameter Kirschner wire (Johnson & Johnson, New Brunswick, NJ, USA; Figure 3F). A 3.5-mm diameter locking compression plate (Johnson & Johnson) was placed on the cranial side of the stifle, then the dynamic compression plate used for temporary fixation was removed. A 3.5-mm diameter string of pearls plate (Veterinary Orthopedic Implants, Saint Augustine, FL, USA) was placed laterally (Figure 3G). The patella was fixed to the medial femoral condyle using a 2.0 mm diameter self-tapping cortical screw (Johnson & Johnson) in lag fashion. The tibial tuberosity that collapsed during the freezing process was supplemented with bioabsorbable artificial bone made of β-Tricalcium phosphate (Olympus Terumo Biomaterials Corp., Tokyo, Japan).

**Figure 4:**
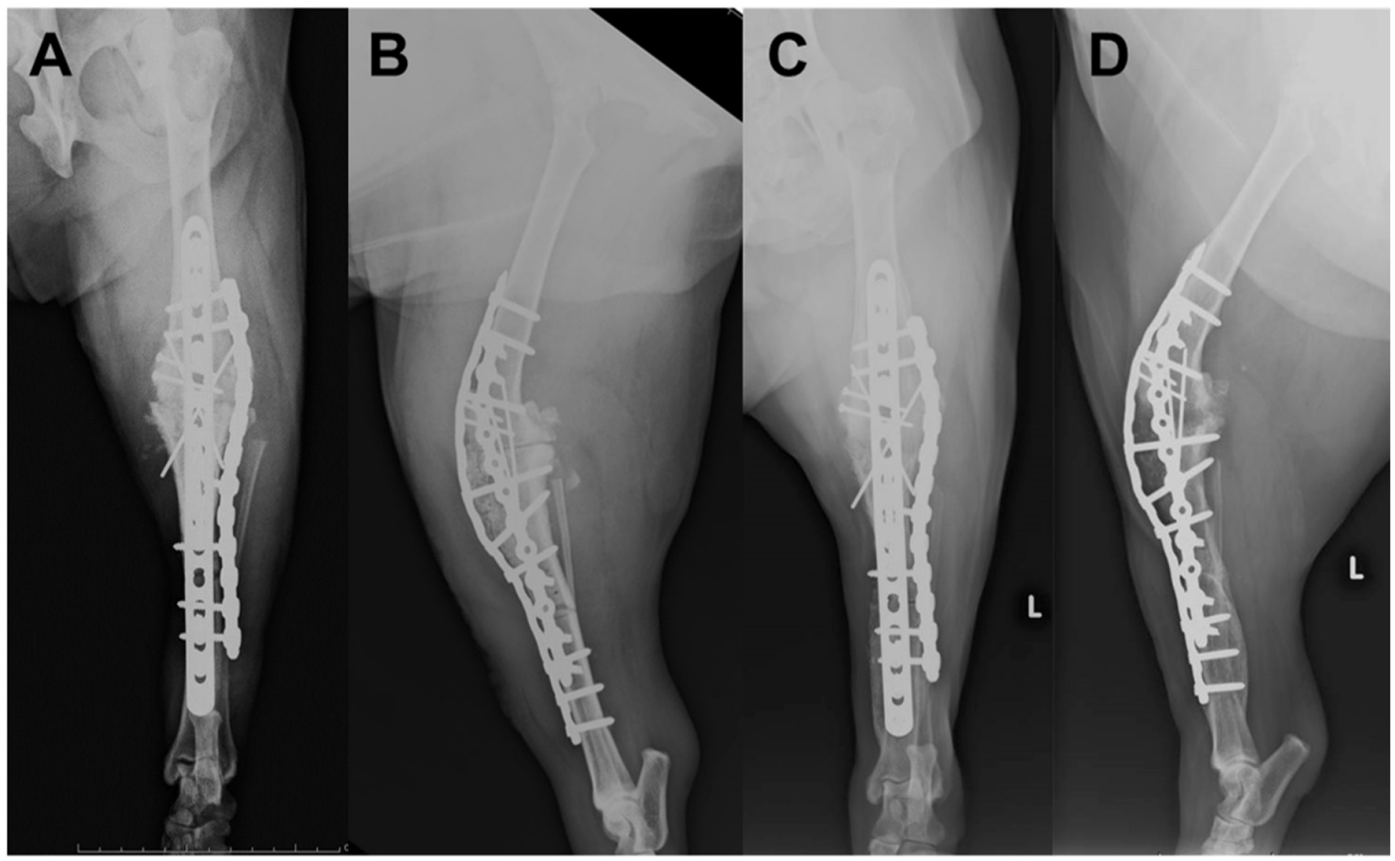
Postoperative radiographs of the left stifle; **(A)** Mediolateral image after surgery; **(B)** Craniocaudal image after surgery; **(C)** Mediolateral image at 20 months postoperatively; **(D)** Craniocaudal image at 20 months postoperatively.

Postoperative radiographs are shown in Figures 4A, B. A fentanyl patch was used for postoperative analgesic management. In addition, a continuous infusion of fentanyl (1–2 μg/kg/h) and medetomidine (0.5–1 μg/kg/h) was administered for 12 h after surgery. After icing, the Robert Jones bandage technique was performed, which was continued for 1 week with daily changes. For antibiotic control, cefmetazole sodium (25 mg/kg, IV) was continued at 12-h intervals for 2 weeks. The tissue taken intraoperatively from the lesion was histologically diagnosed as CSA, which was classified as grade 1 based on previous literature (15). The tissue in the medullary cavity at the distal end of the proximal tibial fragment did not reveal any neoplastic lesions. On the third postoperative day, the weight-bearing ability of the affected limb was observed, and the patient walked around the hospital voluntarily. The weight-bearing ability continued to improve over time, and the patient was discharged from the hospital 2 weeks postoperatively.

Three months postoperatively, the patient was able to run lightly at home. Radiographic images did not show evidence of implant failure, and bone regrowth was observed; however, bone healing was not yet fully achieved. Six months postoperatively, there were no findings of pain or other symptoms of decreased QOL, metastasis, or recurrence. Radiographs showed that the tibiofemoral joint achieved osteosynthesis, and the proximal and distal tibia showed bone activity, but osteosynthesis was incomplete. Currently, 20 months have passed since the surgery, and the general condition and gait are good as reported by the owner. Radiographs showed that the proximal and distal tibia achieved osteosynthesis. Although circumduction gait remained during the postoperative period, the weight-bearing status was good, and the owner was satisfied with the results (Figures 4C, D).

## 3. Discussion

In the present case, CSA of the proximal tibia treated with limb-sparing surgery using frozen autologous bone graft with liquid nitrogen and stifle arthrodesis resulted in a favorable outcome. Postoperatively, although a circumduction gait associated with stifle arthrodesis remained, the patient could maintain a high QOL during the 20-month follow-up period, and the owner was satisfied with the results.

CSA has good prognosis owing to its low metastatic rate and long survival time, with a median survival time of 979 days, even with amputation alone (15). However, the gait changes drastically with amputation. Although hindlimb amputation has been suggested to have a smaller effect on the unaffected limb than forelimb amputation (16), changes in gait and weight-bearing capacity of the forelimb, especially the contralateral forelimb; increase in weight-bearing capacity of the contralateral hindlimb; increase in lateral flexion of the spine; and extension of the lumbosacral joint have been observed after hindlimb amputation (16–18). In the current case, cranial cruciate ligament rupture of the right stifle joint was suspected, and degenerative lumbosacral stenosis was also present. It was predicted that amputation was associated with a risk of postoperative gait disturbance due to the progression of stifle osteoarthritis in the contralateral limb and an increase in the burden of lumbosacral disease. In a previous case of stifle arthrodesis treated using a frozen cortical bone allograft for CSA in a German Shepherd dog, while circumduction gait persisted during the 17-month postoperative follow-up period, the patient could use the affected limb and had an excellent outcome (19). In the present study, the patient could use the affected limb for an extended period (20 months) after surgery, QOL was maintained, and the owner was satisfied with the outcomes. However, there is a lack of research on gait changes caused by stifle arthrodesis; therefore, further long-term follow-up studies on patient gait are needed.

CSA shows high water content and signal intensity on T2WI (20, 21). In the present case, preoperative T2WI imaging was used to localize the tumor. Autoclave and pasteurization methods can be used to reuse bone from tumor sites in humans; however, these techniques require special equipment and strict temperature control, and there are concerns about bone fragility and loss of osteoinductive capacity (11, 22). The liquid nitrogen freezing method used in this study has been shown to kill tumor cells without reducing the strength of normal bone in previous reports (11). However, when the CSA was frozen with liquid nitrogen in the present case, the tumor area became fragile and collapsed. Here, the tumor was localized mainly in the tibial tuberosity. The cortical bone on the caudal aspect, which is the compression side of the stifle joint, was maintained. This may have been one of the factors that allowed the stifle to remain fixed. However, depending on the site of tumor occurrence, the choice of surgical method may need to be considered in patients with CSA. Additionally, preoperative T2WI may be helpful for surgical planning.

In conclusion, limb-sparing surgery with frozen autologous bone grafting using liquid nitrogen can be an alternative to amputation. Thus, the usefulness of this treatment for CSA should be clarified in long-term outcome studies with a large sample size.

## 4. Patient perspective

In this case, a chondrosarcoma developed at the proximal end of the tibia, and joint preservation was not possible. The patient could use the affected limb with joint immobilization, which may be a more favorable outcome than amputation. However, gait changes associated with joint immobilization may affect other orthopedic and neurological conditions, and will require follow-up over time.

## Data availability statement

The original contributions presented in the study are included in the article/supplementary material, further inquiries can be directed to the corresponding author.

## Ethics statement

Written informed consent was obtained from the owner for the participation and publication of this case report.

## Author contributions

MS contributed to study design, acquisition of data, data analysis, interpretation, and drafted manuscript. TN and MM contributed to acquisition of data, data analysis, and interpretation. DY contributed to study design, data analysis, and interpretation. YH contributed to conception of study, study design, acquisition of data, data analysis, and interpretation. All authors also revised and approved the submitted manuscript.
